# A mutant allele of *ζ-carotene isomerase* (*Z-ISO*) is associated with the yellow pigmentation of the “Pinalate” sweet orange mutant and reveals new insights into its role in fruit carotenogenesis

**DOI:** 10.1186/s12870-019-2078-2

**Published:** 2019-11-04

**Authors:** María J. Rodrigo, Joanna Lado, Enriqueta Alós, Berta Alquézar, Orly Dery, Joseph Hirschberg, Lorenzo Zacarías

**Affiliations:** 1 Instituto de Agroquímica y Tecnología de Alimentos (IATA), Consejo Superior de Investigaciones Científicas (CSIC), Calle Catedrático Agustín Escardino 7, 46980 Valencia, Spain; 2 Instituto Nacional de Investigación Agropecuaria (INIA), Salto, Uruguay; 3 Instituto de Biología Molecular y Celular de Plantas (IBMCP) UPV-CSIC, Valencia, Spain; 4 Alexander Silberman Institute of Life Sciences, The Hebrew University of Jerusalem, Jerusalem, Israel

**Keywords:** Carotenoid, ζ-Carotene isomerase, Citrus fruit, gene expression, Mutant, Pigmentation, Ripening

## Abstract

**Background:**

Fruit coloration is one of the main quality parameters of *Citrus* fruit primarily determined by genetic factors. The fruit of ordinary sweet orange (*Citrus sinensis*) displays a pleasant orange tint due to accumulation of carotenoids, representing β,β-xanthophylls more than 80% of the total content. ‘Pinalate’ is a spontaneous bud mutant, or somatic mutation, derived from sweet orange ‘Navelate’, characterized by yellow fruits due to elevated proportions of upstream carotenes and reduced β,β-xanthophylls, which suggests a biosynthetic blockage at early steps of the carotenoid pathway.

**Results:**

To identify the molecular basis of ‘Pinalate’ yellow fruit, a complete characterization of carotenoids profile together with transcriptional changes in carotenoid biosynthetic genes were performed in mutant and parental fruits during development and ripening. ‘Pinalate’ fruit showed a distinctive carotenoid profile at all ripening stages, accumulating phytoene, phytofluene and unusual proportions of 9,15,9′-tri-*cis-* and 9,9′-di-*cis*-ζ-carotene, while content of downstream carotenoids was significantly decreased. Transcript levels for most of the carotenoid biosynthetic genes showed no alterations in ‘Pinalate’; however, the steady-state level mRNA of *ζ-carotene isomerase* (*Z-ISO*), which catalyses the conversion of 9,15,9′-tri-*cis-* to 9,9′-di-*cis*-ζ-carotene*,* was significantly reduced both in ‘Pinalate’ fruit and leaf tissues. Isolation of the ‘Pinalate’ *Z-ISO* genomic sequence identified a new allele with a single nucleotide insertion at the second exon, which generates an alternative splicing site that alters *Z-ISO* transcripts encoding non-functional enzyme. Moreover, functional assays of citrus Z-ISO in *E.coli* showed that light is able to enhance a non-enzymatic isomerization of tri-*cis* to di-*cis*-ζ-carotene, which is in agreement with the partial rescue of mutant phenotype when ‘Pinalate’ fruits are highly exposed to light during ripening.

**Conclusion:**

A single nucleotide insertion has been identified in ‘Pinalate’ *Z-ISO* gene that results in truncated proteins. This causes a bottleneck in the carotenoid pathway with an unbalanced content of carotenes upstream to β,β-xanthophylls in fruit tissues. In chloroplastic tissues, the effects of Z-ISO alteration are mainly manifested as a reduction in total carotenoid content. Taken together, our results indicate that the spontaneous single nucleotide insertion in *Z-ISO* is the molecular basis of the yellow pigmentation in ‘Pinalate’ sweet orange and points this isomerase as an essential activity for carotenogenesis in citrus fruits.

## Background

*Citrus* fruit is the main category of tree fruit crops in the world, with a total production of 124 MT. Sweet orange *(Citrus sinensis),* the most cultivated worldwide, accounts for 62% of the total citrus fruits production (FAOSTAT, 2017). As in many other fruits, the pigmentation of sweet orange is one of the most important quality traits, which is due to the content and composition of carotenoids [1]. Carotenoids constitute a large family of isoprenoid-compounds with more than 1182 different structures [2, 3]. Carotenoids are synthetized by all photosynthetic organisms in plastids and cover essential roles in the photosystems, as photoprotective molecules, and are the precursors of phytohormones abscisic acid (ABA) and strigolactones, and other signalling molecules [4].

The carotenoid biosynthetic pathway is well established in model plants and agronomic important crops, being tomato the preferred model for carotenogenic studies in fruit [5–7]. In the genus *Citrus* the high diversity in fruit pigmentation among different species and varieties is based on their specific carotenoid complements [8–10]. In mature fruit of sweet oranges the qualitative carotenoid composition in flavedo and pulp is similar but flavedo contains about 4 to 10-fold more carotenoids, ranging the total content from 50 to 150 μg g^− 1^ fresh weight (FW) in the flavedo and 5–25 μg g^− 1^ FW in the pulp. The carotenoid profile in mature sweet orange fruit tissues displays more than 80% of the total content of β,β-xanthophylls, being 9-*cis*-violaxanthin the predominant carotenoid [11–17]. Other β,β,xanthophylls such as antheraxanthin, β-cryptoxanthin, neoxanthin and zeaxanthin, the carotene phytoene and the C30-apocarotenoid β-citraurin, are present in variable amounts depending on the fruit tissue and the cultivar [18, 19]. Minor concentrations of phytofluene, α-, β- and ζ-carotene, and other xanthophylls have been reported [20].

**Fig. 1:**
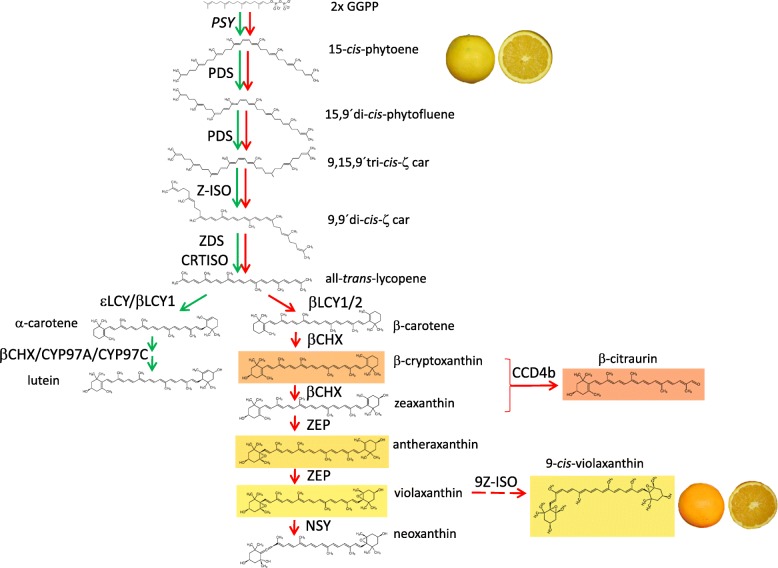
Schematic representation of carotenoid biosynthetic pathway in sweet orange (*Citrus sinensis*) fruit indicating main enzymes involved in carotenoid compositional changes during ripening. The predominant flux in immature and full ripe fruit tissues is indicated in green and red arrows, respectively. The discontinuous arrow indicates a enzymatic step not yet characterized. The structure of the main carotenoids involved in sweet orange fruit pigmentation is background colored. Representative pictures of ‘Pinalate’ sweet orange mutant and its parental genotype ‘Navelate’ are positioned in the pathway close to the most abundant carotenoid in mature fruit tissues. *PSY*, phytoene synthase; *PDS*, phytoene desaturase; *Z-ISO*, 15-*cis*-ζ-carotene isomerase; *ZDS*, ζ-carotene desaturase; *CRTISO*, lycopene isomerase; *βLCY1*, lycopene β-cyclase 1; *βLCY2*, chromoplast-specific lycopene β-cyclase2; *εLCY*, lycopene ε-cyclase; *CYP97A* and *CYP97C*, heme-containing cytochrome P450 hydroxylases; *βCHX*, non-heme β-carotene hydroxylase; *ZEP*, zeaxanthin epoxidase; *NSY*, neoxanthin synthase; *CCD4b*, carotenoid cleavage dioxygenase type 4; *9-Z-ISO*, 9-Z-violaxanthin isomerase. *GGPP*, Geranyl geranyl pyrophosphate. Carotenoid structure images were obtained from http://carotenoiddb.jp/ [2]

Changes in carotenoids during fruit ripening of sweet orange are primarily regulated by the transcriptional level of the main structural carotenoid biosynthetic genes (Fig. 1) [8–10, 12, 21, 22]. The flavedo of immature sweet orange shows a carotenoid profile characteristic of chloroplastic-containing tissue being lutein the most abundant carotenoid followed by α- and β-carotenes, and all-*trans*- violaxanthin, neoxanthin and zeaxanthin [8, 11, 12, 23–25]. At this green or immature stage, the expressions of phytoene synthase (*PSY*), phytoene desaturase (*PDS*) and ζ-carotene desaturase (*ZDS*) genes, involved in the early biosynthetic steps of the pathway (Fig. 1), have been reported to be relatively low [11, 12]. During the onset of color break the expression of most of the genes involved in the upstream reactions, particularly *PSY*, is highly up-regulated enhancing the flux of the pathway [9, 11, 12, 15, 21].

In plants, the action of two isomerases, 15-*cis*-ζ-carotene isomerase (Z-ISO) [26, 27] and lycopene isomerase (CRTISO) [28], are needed to obtain all-*trans-*lycopene, the cyclases substrate (Fig. 1). The expression of *CRTISO* in different *Citrus* species does not correlate with the β,β-xanthophylls accumulation [12], and the characterization of citrus *Z-ISO* is restricted to pummelo (*Citrus maxima*) [29].

The lycopene ε-cyclase gene, *εLCY*, is down-regulated during ripening of sweet orange fruit having a prominent role redirecting the flux of the pathway to the β,β-branch (Fig. 1) [11]. In citrus fruit the cyclization of lycopene to produce β-carotene is catalyzed by two different enzymes β-lycopene cyclase (βLCY). The *βLCY1* displays a constitutive expression in sweet orange fruit tissues during ripening, while *βLCY2* is only expressed in chromoplastic tissues and parallels with the accumulation of β,β-xanthophylls [14, 30]. Subsequently in the pathway, four enzymes have been reported to be involved in α- and β-carotene hydroxylation in citrus to produce xanthophylls: CitHYb/βCHX, CitCYP97A, CitCYP97B, and CitCYP97C; however, transcriptional and functional studies suggest that only βCHX plays a relevant role in β,β-xanthophyll biosynthesis in citrus fruits [31].

The last enzyme characterized in the plant carotenoid biosynthetic sequence is the 15- *cis*-ζ-carotene (Z-ISO) which catalyzes the *cis-trans* isomerization of central double bond (15–15′) of the 9,15,9′-tri-*cis*-ζ-carotene, product of PDS, to 9,9′-di-*cis*-ζ-carotene, substrate to ZDS [26, 27, 32]. The Z-ISO is an integral membrane protein that carries a heme-b cofactor that undergoes redox-dependent ligand switching [27]. The Z-ISO in vivo function was revealed by the identification of Arabidopsis *zic1* and maize *y9* mutants [26, 32] and tomato *Z-ISO*-VIGS-silenced fruits [33]. All defective Z-ISO plants showed the characteristic accumulation of 9,15,9′-tri-*cis*-ζ-carotene in addition to a noteworthy increase in phytoene and phytofluene [32, 33]. Interestingly, the function of Z-ISO can be partially compensated by light since 15–15’*cis-trans* photoisomerization of 9,15,9′-tri-*cis*-ζ-carotene occurs [26, 33].

A key strategy to investigate *Citrus* carotenogenesis is the use of the natural genetic diversity in fruit pigmentation among the large collections of species and spontaneous mutants [16, 34]. The best characterized sweet orange mutants with distinctive fruit coloration are the red-fleshed mutants ‘Cara Cara’ and ‘Hong Anliu’. Both accumulate lycopene in the pulp, a carotene absent in ordinary sweet oranges [13, 16, 23] and contain unusual elevated amounts of the upstream carotenes phytoene and phytofluene, and reduced levels of downstream β,β- xanthophylls [13, 23, 35]. Transcriptional and metabolomic studies suggest an alteration in βLCY activity in both mutants, either in βLCY1 and/or in the chromoplast-specific βLCY2 [36, 37] and in the MEP pathway genes [13].

The sweet orange ‘Pinalate’ is a spontaneous bud mutant from ‘Navelate’ genotype with distinctive yellow fruits due to a significant reduction in β,β-xanthophylls [38]. The biochemical data suggest an alteration in ‘Pinalate’ fruit related to ZDS desaturase or ZDS-associated factors [38, 39]. ‘Pinalate’fruits also display reduced levels of the phytohormone ABA, a downstream product of carotenoid metabolism [38]. Due to this singularity, ‘Pinalate’ mutant has been used as experimental tool to investigate the perception and signaling components of ABA in citrus [40] and their role in fruit dehydration and other postharvest disorders [41–44]. Moreover, ‘Pinalate’ was used to explore the relationship between plastid ultrastructure and carotenoid composition in citrus fruits [39] and to investigate the bioaccessibility of phytoene and phytofluene in orange juice [45]. However, the molecular basis of the ‘Pinalate’ mutation that causes the partial blockage in the biosynthesis of carotenoids and the yellow phenotype remains elusive and is the main objective of the present work. The comparative analysis of carotenoids and transcriptional profile of main carotenoid biosynthetic genes in fruit and vegetative tissues of ‘Pinalate’ and its parental ‘Navelate’ suggested an alteration in the Z-ISO activity. Thus, the isolation of *Z-ISO* gene and itsfunctional characterization from mutant and wild-type showed that ‘Pinalate’ harbours a mutated *Z-ISO* allele in heterozygous state that generates non-functional proteins.

## Results

### The yellow ‘Pinalate’ sweet orange mutant shows altered carotenoid profile in fruit tissues during ripening and reduced carotenoid content in leaves

**Fig. 2:**
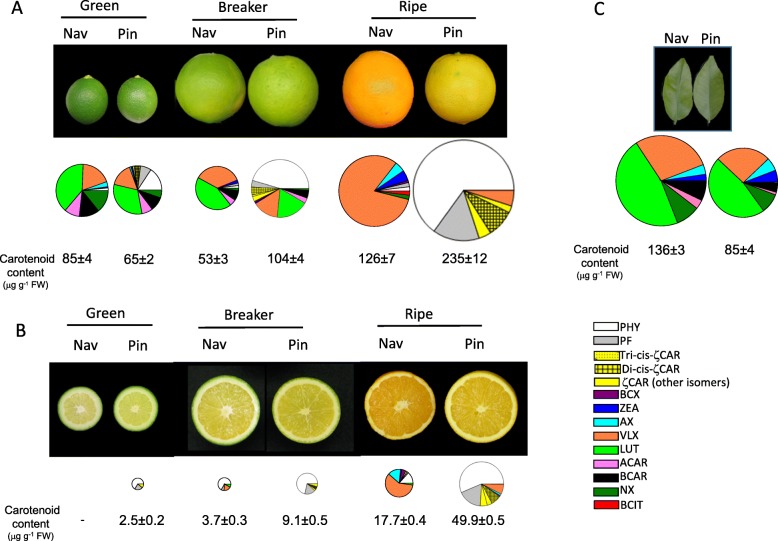
External (**a**) and internal (**b**) aspect of parental ‘Navelate’ (Nav) and mutant ‘Pinalate’ (Pin) fruits at immature green, breaker and full ripe stage, and young leaves (**c**). Graph pies represented the distribution of individual carotenoids, as percentage of total carotenoid content, in the corresponding Nav and Pin samples, and data below indicate the total carotenoid content (μg g^− 1^ FW). Note that pie graphs are proportional to the total carotenoid content

Changes in carotenoid content and composition were evaluated in flavedo and pulp of ‘Pinalate’ fruits at three ripening stages: immature green, breaker and fully ripe, and compared with those of its wild-type ‘Navelate’ (Fig. 2). In immature green flavedo the total carotenoid content in ‘Pinalate’ was about 25% lower (65 μg g^− 1^) than in parental fruits (85 μg g^− 1^) and differences in individual carotenoids were identified. As in parental fruit, the main carotenoid in ‘Pinalate’ green flavedo was the β,ε-xanthophyll lutein (19.5 μg g^− 1^ which represents about 30%) containing also 17 μg g^− 1^ of β,β-xanthophylls (25%) (neoxanthin and violaxanthin) as well as α- and β-carotene (14.3 μg g^− 1^). Interestingly, ‘Pinalate’ green flavedo contained more than 25% of the upstream carotenes (phytoene, phytofluene and ζ-carotene) which were barely detectable in the parental fruit. In agreement with previous data [46], different ζ-carotene isomers were detected in ‘Pinalate’ and in the context of this study it is important to mention that at this green stage the presence of 9,15, 9’tri-*cis*-ζ-carotene isomer was not detected and the predominant isomer was 9, 9’di-*cis*-ζ-carotene (2.7 μg g^− 1^) (Fig. 2a). At the breaker stage, flavedo of both genotypes exhibited a light green coloration with yellow patches indicative of chlorophylls degradation and the initiation of chloroplasts to chromoplasts transition (Fig. 2a). Flavedo of ‘Pinalate’ fruit at this stage contained almost double carotenoid content (104 μg g^− 1^) than those of ‘Navelate’ (53 μg g^− 1^), and while in ‘Pinalate’ this amount involved an 80% increment over immature green fruits, contrastingly in ‘Navelate’ represented a 40% reduction. The carotenoid composition in the peel of both genotypes was different at breaker stage. In wild-type the profile was similar to green fruit but an increase in the proportion of β,β-xanthophylls was already evident, indicating the enhancement of carotenogenesis associated with fruit ripening. In ‘Pinalate’ the content of phytoene (46.8 μg g^− 1^) and of ζ-carotene (9.4 μg g^− 1^) was increased by 3-fold compared to green flavedo and, more importantly, the presence of 9,15,9’tri-*cis*-ζ-carotene was detected (1.2 μg g^− 1^). At ripe stage, total carotenoid content in the flavedo of ‘Pinalate’ almost doubled that of the wild-type (235 and 126 μg g^− 1^, respectively) and the composition was strikingly different. The flavedo of ‘Navelate’ contained more than 90% of β,β-xanthophylls, with 105.1 μg g^− 1^ of violaxanthin and 14.7 μg g^− 1^ of other β,β-xanthophylls (zeaxanthin, anteraxanthin and β-cryptoxanthin), while upstream carotenes, including phytoene and phytofluene, accounted for 5.2 μg g^− 1^ and ζ-carotene was not detected. In ‘Pinalate’, phytoene and phytofluene were overrepresented with concentrations of 153 and 36 μg g^− 1^, respectively, and several isomers of both carotenes were identified. Six different isomers were detected for ζ-carotene (Additional file 1: Figure S1), with concentrations of 5.1 and 20.3 μg g^− 1^ for 9,15,9’tri-*cis* and 9,9’di-*cis*, respectively, and 8.9 μg g^− 1^ for all other ζ-carotene isomers. Violaxanthin was the only xanthophyll identified in the flavedo of ‘Pinalate’ and its concentration was 11.8 μg g^− 1^ which is approximately 10-fold lower than in ‘Navelate’.

In the pulp of both genotypes, significant differences in carotenoid content and composition were obvious since green immature stage (Fig. 2b). Carotenoids concentration in the pulp of green ‘Navelate’ fruit was negligible while in ‘Pinalate’ minor amounts of phytoene (1.8 μg g^− 1^), phytofluene (0.4 μg g^− 1^) and ζ-carotene (0.3 μg g^− 1^) were detected. At breaker stage, ‘Navelate’ pulp displayed low concentration of carotenoids being phytoene the main carotene (2.6 μg g^− 1^) but also contained a significant proportion of β,β-xanthophylls (0.8 μg g^− 1^), particularly violaxanthin and anteraxanthin. In ‘Pinalate’ there was almost a 4-fold increase in the total carotenoid content compared to green stage with a carotenoid profile similar to green stage. Carotenoid content in the pulp of mature ‘Navelate’ fruit was 5-fold higher than at breaker, and was predominantly composed by β,β-xanthophylls (13.3 μg g^− 1^) but also phytoene (3.5 μg g^− 1^), phytofluene (0.5 μg g^− 1^), and traces of 9,9’di-*cis*-ζ-carotene and β-carotene. In ripe ‘Pinalate’ pulp the concentration of total carotenes increased by 5-fold compared to breaker stage and it was almost 3-fold higher than in ‘Navelate’. Phytoene was the predominant carotenoid (27.7 μg g^− 1^) representing more than 50% of the total content, followed by phytofluene (8.7 μg g^− 1^) and ζ-carotene (3.7 μg g^− 1^ of 9,9’di-*cis* isomer and 1.8 μg g^− 1^ of 9,15,9’tri-*cis*). The concentration of β,β-xanthophylls was more than 2-fold lower (5.3 μg g^− 1^) than in ‘Navelate’ with a major proportion of violaxanthin. Representative chromatograms illustrating the marked differences in carotenoid profile between the pulp of ‘Pinalate’ and Navalete ripe fruits are shown in Additional file 2: Figure S2.

The carotenoid composition in young leaves of both genotypes was very similar and typical of chloroplastic tissues, with a predominance of lutein and other β,β-xanthophylls (violaxanthin, neoxanthin, zeaxanthin) and α- and β-carotene (Fig. 2c). However, the carotenoid content was 40% lower in ‘Pinalate’ leaves compared to parental and, in contrast with fruit tissues, there was no accumulation of upstream carotenes. The chlorophyll content was also reduced by half in mutant leaves (545 μg g^− 1^) compared to the parental ones (1146 μg g^− 1^).

In the context of this study, it is relevant to mention that neither lycopene nor neurosporene were detected in either mutant and wild-type fruit or leaf extracts.

### Light growing conditions affect carotenoid profile of ‘Pinalate’ fruit peel

**Fig. 3:**
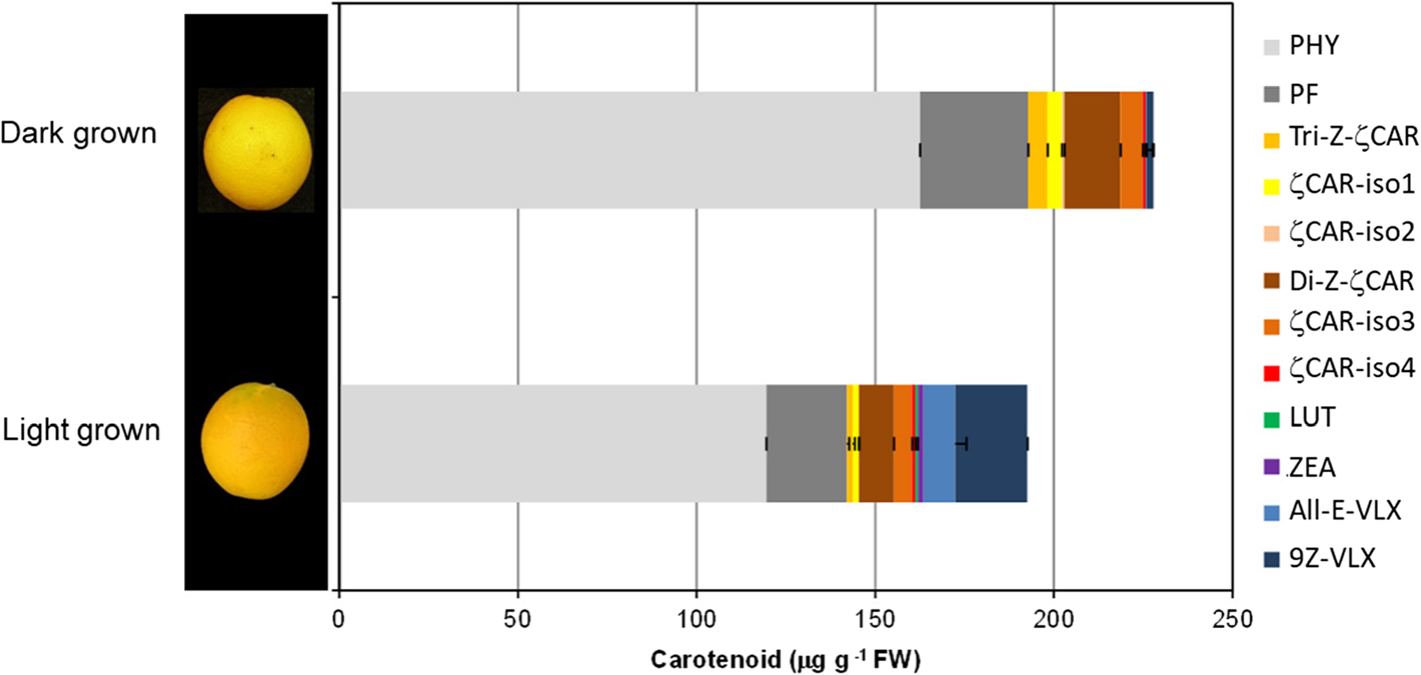
Effect of light intensity and dark growing conditions on carotenoid content and composition in peel of ‘Pinalate’ mutant fruit. ‘Pinalate’ fruits grown in dark conditions were covered at immature green stage with black plastic bags (bottom-end open) and harvested in full ripe stage. Light exposed fruits were harvested at full ripe stage from the South oriented side of the canopy tree to maximize the effect of light exposure

The abnormal accumulation of colorless carotenes and ζ-carotene isomers in ‘Pinalate’ fruit tissues suggests a partial blockage either in ZDS or Z-ISO activity. It has been reported that Z-ISO activity, but not ZDS, can be partially compensated by light, and under dark growing conditions the biochemical blockage may be intensified [33]. In order to investigate the effect of light/dark conditions on ‘Pinalate’ carotenoids profiling, we covered ‘Pinalate’ fruits with black plastic at immature green stage and allowed them to develop and ripe under darkness until harvest [46]. As control, ‘Pinalate’ fruits directly exposed to sunlight from the external tree canopy were selected. Light and dark-grown fruits were harvested at the ripe stage and the carotenoid profile was determined in the flavedo. At harvest, the external color of the fruits grown under both conditions was different: light-grown fruit displayed a pale-orange coloration while dark-grown fruits where completely light-yellow with any signals of orange tint (Fig. 3). Total carotenoid content was higher in dark-grown (227 μg g^− 1^) than in light-exposed fruit (195 μg g^− 1^) and differences in carotenoid profile were also observed. Dark-grown ‘Pinalate’ flavedo contained a larger proportion (84% of the total carotenoids) and content of phytoene and phytofluene (191 μg g^− 1^), and reduced xanthophylls (2.2 μg g^− 1^ accounting for 0.97% of the total) compared to light-grown which contained 74% of colorless carotenes (143 μg g^− 1^) and 16% of β,β-xanthophylls (33 μg g^− 1^) (Fig. 3). Interestingly, the content and the ratio of 9,15,9’tri-*cis*:9,9’di-*cis* ζ-carotene isomers was also affected by light conditions. Thus, whereas in light-grown fruits the ratio tri-*cis*:di-*cis* was 0.17 in dark-grown increased up to 0.34. Moreover, the concentration of tri-*cis* was 3-fold higher in the flavedo of dark-grown compared to that of light-exposed fruits.

### Transcriptional profiles of main carotenoid biosynthetic genes in ‘Pinalate’ do not correlate with its carotenoid composition

**Fig. 4:**
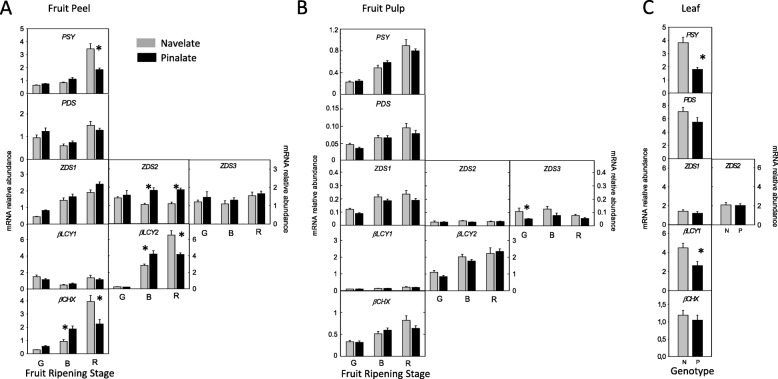
Carotenoid biosynthetic gene expression in peel (**a**) and pulp (**b**) of ‘Navelate’ (grey bars) and ‘Pinalate’ mutant (black bars) fruits at green (G), breaker (B) and full ripe (R) stage, and (**c**) in young leaves of ‘Navelate’ and ‘Pinalate’ . Asterisks indicate significant differences between ‘Navelate’ and ‘Pinalate’ samples (*p* ≤ 0.01). Expression of *ZDS3* and *βLCY2* was not detected in leaves. *PSY*, phytoene synthase; *PDS*, phytoene desaturase; *ZDS1,2 and 3*, ζ-carotene desaturase; *βLCY1*, lycopene β-cyclase 1; *βLCY2*, chromoplast-specific lycopene β-cyclase2; *βCHX*, non-heme β-carotene hydroxylase

Since carotenoid composition in ‘Pinalate’ fruit suggests an impairment in the pathway flux toward the *β,β-*xanthophylls production, we performed a comparative transcriptional analysis of main genes involved in their synthesis: *PSY, PDS, ZDS1, ZDS2* and *ZDS3*, *βLCY1* and *βLCY2*, and *βCHX*, in flavedo, pulp and leaves of mutant and wild-type (Fig. 4). In both genotypes *PSY, ZDS1*, *βLCY2* and *βCHX* genes were upregulated in flavedo and pulp during ripening while *PDS* gene was almost constitutively expressed in pulp. The expression of *PDS* in flavedo, and *ZDS2, ZDS3* and *βLCY1* in both fruit tissues, did not show clear trends or were constitutive (Fig. 4a, b). In general, the pattern and relative expression level for most of the genes was similar in ‘Pinalate’ and ‘Navelate’, and only some genes showed differences between genotypes: *PSY*, *βLCY2* and *βCHX* transcript levels were significantly lower in flavedo of mature ‘Pinalate’ fruits, and expression of *βCHX* (breaker stage) and *ZDS2* (ripe stage) were higher in ‘Pinalate’. The relative transcript level of additional genes such as *PSY2* [9], *CRTISO*, *CYP97A* and *ZEP* were also determined in ‘Pinalate’ and ‘Navelate’ flavedo at breaker stage and no significant differences between both genotypes were detected (data not shown). In the pulp, the only difference between ‘Pinalate’ and ‘Navelate’ gene transcript levels was the reduced expression of *ZDS3* in ‘Pinalate’ green fruits. Altogether, variations in the transcript levels between ‘Pinalate’ and ‘Navelate’ fruit tissues do not explain by themselves the differences in carotenoid composition between both genotypes.

Since young leaves of ‘Pinalate’ and ‘Navelate’ did not show differences in carotenoid profile but content was reduced by half in the mutant, we also analysed the relative expression levels of carotenoid biosynthetic genes (Fig. 4c). The expression of *PSY* and *βLCY1* was approximately 50% lower in ‘Pinalate’ but other genes showed similar transcript levels in both genotypes (Fig. 4c). No expression of *ZDS3* and *βLCY2* genes was detected in leaves samples.

### ‘Pinalate’ sweet orange mutant harbours a new *Z-ISO* allele with a single nucleotide insertion

**Fig. 5:**
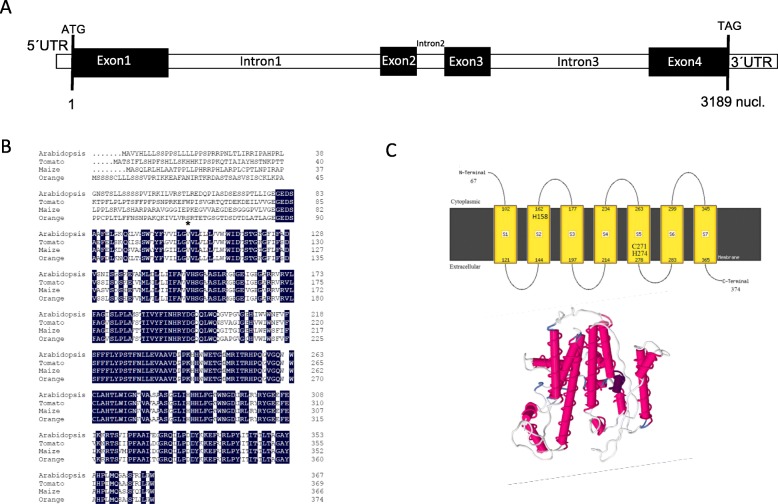
Sweet orange *Z-ISO* gene and protein structures. **a** Structure of sweet orange *Z-ISO* gene with four exons (black) and three introns (white). **b** Alignment of Z-ISO proteins from sweet orange (KDO50569.1), Arabidopsis (NP563879.1), maize (NP_001132720.1) and tomato (XP_004252966.1). Numbers on the left denote the number of amino acid residues and identical for all sequences in a given position are in black background. The end of predicted a plastid transit peptide marked with an asterisk. **c** Sweet orange Z-ISO topology predicted by MEMSAT3 and tridimensional modelling of Z-ISO (PPMserver) by using iTASSER. Transmembrane α-helix S1 to S7 are represented in yellow and pink in the 2D and 3D-models, respectively

The massive accumulation of early carotenes in ‘Pinalate’ fruit tissues together with the abnormal presence of 9,15,9’tri-*cis*-ζ-carotene without evident correlation with transcriptional profile of main carotenoid biosynthetic genes, and the partial rescue of flavedo wild-type phenotype when mutant fruits were exposed to light, strongly suggest a deficiency in the 15-*cis*-ζ-carotene isomerase, Z-ISO. In order to explore this hypothesis we identified the sweet orange *Z-ISO* gene by BLAST search in the *Citrus sinensis* v1.1 genome assembly in Plant Comparative Genomics portal Phytozome (https://phytozome.jgi.doe.gov/) using the Z-ISO from Arabidopsis (NP563879.1). A single gene was identified (orange1.1g017272m.g) with a length of 3189 nucleotides and similar genomic structure to Arabidopsis and maize homologues [26] (Fig. 5a). Sweet orange *Z-ISO* gene contains four exons and three introns encoding a predicted protein of 374 amino acids. The alignment of orange Z-ISO protein with homologues from Arabidopsis, maize and tomato showed a high degree of identity (76–71% at protein level) and most of the variability was detected at the N-terminus which corresponds with the plastid transit peptide predicted by ChloroP1.1 (Fig. 5b). A model prediction of 3D structure of the sweet orange ZISO was generated in the PPMserver by using I-TASSER [47]. For modelling, two oxidoreductases: the integral membrane sterol reductase from *Methylomicrobium alcaliphilum* (Acc. PBD: 4QUV) and phenol hydroxylase-Regulatory Protein Complex from *Pseudomonas* (Acc. PBD: 2INP) were used [27]. The topology of sweet orange Z-ISO was also investigated by using MEMSAT3 [48]. The 3D model and topology analysis for orange Z-ISO showed a structure with seven transmembrane α-helices (S1 to S7) (Fig. 5c) suggesting that is an integral membrane protein as reported for maize Z-ISO [27]. The mechanistic study of maize Z-ISO showed that H150 (transmembrane domain 2) and, C263 and H266 (transmembrane domain 5) are critical residues for isomerization involved in the cofactor (heme) binding and reversible heme ligation. In sweet orange Z-ISO homologous residues were identified in transmembrane domains S2 (H158) and S5 (C271 and H274) (Fig. 5b).

**Fig. 6:**
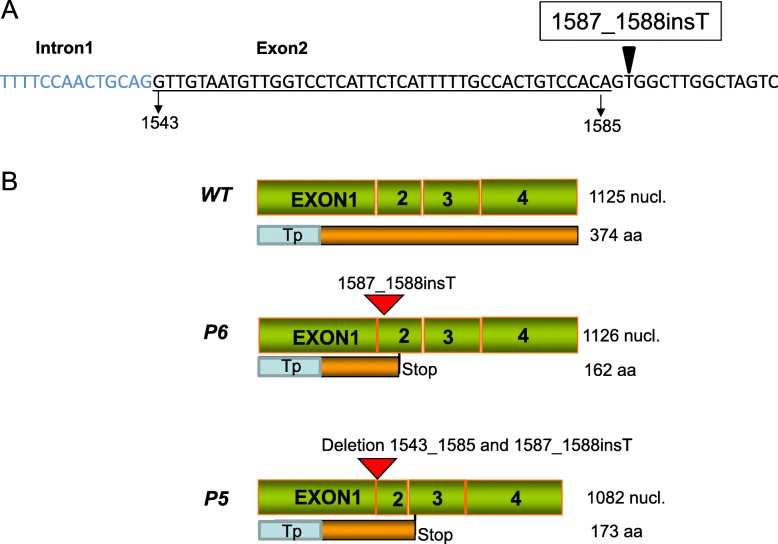
Detail of genomic sequence of intron1 (blue letter) and exon2 (black) *Z-ISO* isolated from ‘Navelate’ and displaying the T insertion in exon2 identified in ‘Pinalate’ mutant (**a**). The underlined sequence in exon2 is deleted as a result of abnormal splicing in a ‘Pinalate’ transcript variant. (**b**) Structures of *Z-ISO* transcripts isolated from ‘Navelate’ and ‘Pinalate’. *Z-ISO WT* transcript encodes a 374 amino acid protein and was isolated from ‘Navelate’ and ‘Pinalate’ fruits; *Z-ISO P5* and *P6* sequences were isolated from ‘Pinalate’. The *Z-ISO P6* contains the T insertion in exon 2 (position 1587_1588) and *Z-ISO P5* transcript has a 42 bp deletion at the junction of intron1-exon2 plus T insertion in exon 2 (position 1587_1588). Alterations in *P5* and *P6* transcripts sequences resulted in premature stop codons and generated truncated proteins. Tp, Predicted plastid transit peptide

Genomic sequencing of *Z-ISO* from wild-type ‘Navelate’ revealed a unique sequence for all clones isolated and identical to orange1.1g017272m.g available at the *Citrus sinensis* genome. By contrast, in ‘Pinalate’ mutant two different genomic sequences were isolated, one identical to wild-type and a second allele with a T insertion in the exon 2 at position 1588 (accession number MN417949) (Fig. 6a). Recently, it is has been reported that *Z-ISO* is a single copy gene in citrus [49] which is also compatible with information obtained from *Citrus sinensis* genome database; therefore, the presence of two different alleles indicates heterozygosity at *Z-ISO* locus in ‘Pinalate’.

The cDNA sequencing of *Z-ISO* from parental ‘Navelate’ sweet orange showed a single sequence with an ORF of 1125 nucleotides (Fig. 6b). PCR amplification of full-length *Z-ISO* cDNAs from ‘Pinalate’ samples (flavedo and pulp tissues at breaker and ripe stages) revealed the presence of three *Z-ISO* transcript variants in the mutant (Fig. 6b). Out of 71 independent cDNA clones from ‘Pinalate’, 36 corresponded to wild-type sequence (*WT*), 29 were identical to wild-type plus a nucleotide insertion (T) in exon 2 at position 1587_1588 (*P6*), and 6 sequences (*P5*) showed a deletion of the first 42 nucleotides of exon 2 (positions 1543–1585) in addition to the T insertion identified in *P6* (Fig. 6a,b). Thus, approximately 50% of the ‘Pinalate’ *Z-ISO* transcripts showed an altered sequence (Fig. 6b): the *P5* resulted in a shorter sequence (1082 nucleotides) and *P6* contained one extra nucleotide compared to the wild-type. Both *P5* and *P6* give frameshift mutations with premature stop codons at 523 and 487 nucleotides and truncated proteins with 177 and 163 amino acids, respectively (Fig. 6b). The 42 nucleotides deletion in *P5* transcripts can be explained by an aberrant splicing at the intron 1-exon 2 caused by the T nucleotide insertion. The sequence (CAGTTG) generated by T insertion in ‘Pinalate’ *Z-ISO* allele (Fig. 6a) highly resembles the intron 1-exon 2 junction (CAGGTTG) and might create a novel acceptor splice site skipping the first 42 nucleotides of exon 2.

The cDNA sequence data suggest that ‘Pinalate’ is heterozygous in the *Z-ISO* locus. This possibility was confirmed by direct sequencing genomic DNA of *Z-ISO* from ‘Pinalate’. The nucleotide sequencing trace up to the T insertion site was unblemished indicating that is identical in both alleles. From the T insertion and on, the sequence trace became blurred due to a frameshift in one allele (Additional file 3: Figure S3).

### The *Z-ISO* encodes a bona fide 15-*cis*-ζ-carotene isomerase but mutant variants (*P5* and *P6*) are not functional

**Fig. 7:**
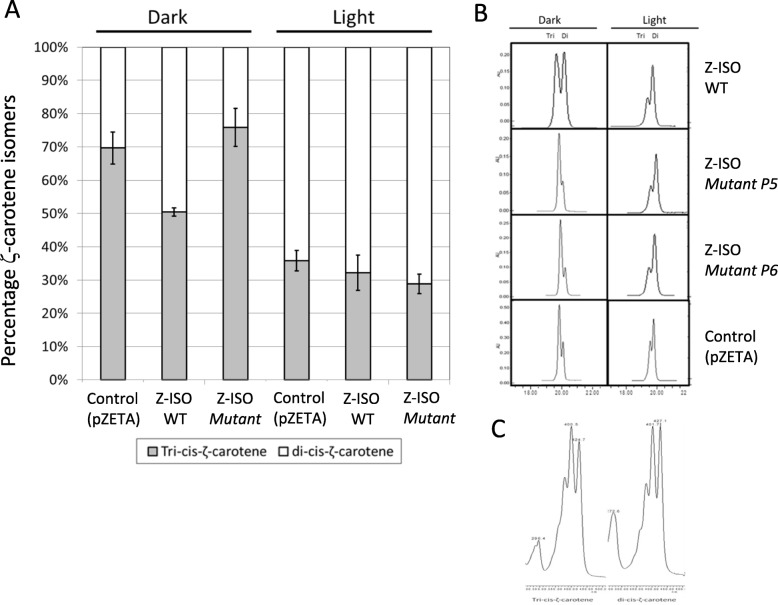
**a** Functional analysis of *Z-ISO* from *WT*, *P5* and *P6* cDNAs in *E. coli* grown under dark and light conditions. *E. coli* with pZETA alone was used as control. Carotenoid composition in *E. coli* cells was determined by HPLC-PAD as described. **b** Representative chromatograms for *Z-ISO WT*, Mutant (*P5* and *P6)* sequences and control (pZETA) under dark and light conditions. **c** Spectra of 9,15,9′-tri-*cis*-ζ-carotene and 9, 9′-di-*cis*-ζ-carotene

The 15-*cis*-ζ-carotene isomerization activity of *Z-ISO* variants was tested in *E. coli* cells carrying the pZETA vector that produced 9,15,9’tri-*cis*-ζ-carotene. To that end, the full-length cDNAs of *Z-ISO* isolated from wild-type ‘Navelate’ (*WT*) and ‘Pinalate’ mutant, (variants P5 and P6) were cloned in the plasmid vector pGEM-T. *E. coli* cells carrying pZETA alone (control), pZETA together with WT or mutant *Z-ISO* cDNAs, were selected following transfection to *E. coli* cells and selection with the appropriate antibiotics. Carotenoid composition in the bacteria grown in suspension cultures was analyzed following incubation in dark or light conditions. *E. coli* cells with pZETA produced 9,15,9’tri-*cis*-ζ-carotene and 9,9’di-*cis*-ζ-carotene at a ratio of 1:2, respectively (Fig. 7). In the presence of wild-type Z-ISO this ratio was changed to 1:1, but the mutant Z-ISO (P5 or P6) was inactive. Similar results were obtained when Z-ISO cDNAs from ‘Pinalate’ (P5 or P6) and ‘Navelate’ were expressed in *E. coli* with the plasmid pPROLYCOPENE (Additional file 4: Figure S4). These results demonstrate that citrus Z-ISO codes for a bona fide 15-*cis*-ζ-carotene isomerase and both cDNA variants from ‘Pinalate’ abolished Z-ISO activity.

The presence of di-*cis*-ζ-carotene in *E. coli* with pZETA alone may have resulted from a spontaneous chemical isomerization of the 15-*cis* double bond. This possibility is supported by the finding that when the *E. coli* cell cultures were exposed to dim white light (50 μmol photons m^− 2^ s^− 1^), the non-enzymatic conversion of tri-*cis* to di-*cis*-ζ-carotene increased even more than the enzymatic process (Fig. 7a, b).

However, conversion of tri-*cis* to di-*cis* with P5 and P6 mutant gene products in dark conditions was similar to empty vector pZETA, indicating no Z-ISO function for P5 and P6 while the functionality of WT Z-ISO sequence was demonstrated by a 25% increase in the proportion of di-*cis*-ζ-carotene (Fig. 7a).

### Expression level of *Z-ISO* is significantly down-regulated in ‘Pinalate’ mutant

**Fig. 8:**
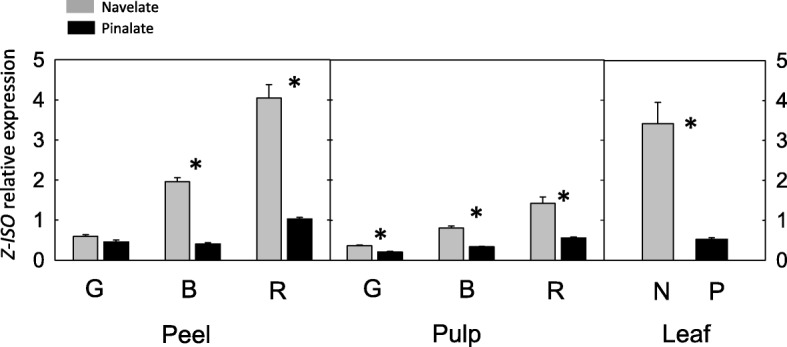
Expression of *Z-ISO* gene in ‘Navelate’ (grey bars, N) and ‘Pinalate’ mutant (black bars, P) in peel and pulp from fruits at green (G), breaker (B) and full ripe (R) stage, and in young leaves. Asterisks indicate significant differences between ‘Navelate’ and ‘Pinalate’ samples (p ≤ 0.01)

The expression of *Z-ISO* gene was analyzed during fruit ripening and in young leaves from parental ‘Navelate’ and mutant ‘Pinalate’. In parental fruit tissues a significant up-regulation in *Z-ISO* expression occurred during ripening, more noticeable in flavedo than in pulp (Fig. 8). In ‘Navelate’ ripe fruit compared to immature stage, the expression of *Z-ISO* was almost 8 and 3-fold higher in flavedo and pulp, respectively (Fig. 8). By contrast, the *Z-ISO* expression only exhibited a moderate increase in ‘Pinalate’ fruit tissues during ripening. As a result, in mutant flavedo, pulp and leaves the *Z-ISO* expression was 3.8-, 2.5- and 5-fold lower than in wild-type, respectively, and only immature green peel of ‘Pinalate’ showed similar expression than parental (Fig. 8).

## Discussion

The coloration of sweet orange is due to the carotenoid complement and is one of the main quality attributes with a major impact in consumers’ acceptance. In addition, carotenoids in the flesh influence nutritional and health-related properties associated with sweet orange intake [1]. Research on carotenoid metabolism in citrus fruit has been an active field and most of the structural enzymes involved in the biosynthetic pathway have been functionally characterized [8–10, 21].

The ‘Pinalate’ sweet orange was originally identified as a bud mutant from ‘Navelate’ in a commercial orchard in San Pedro del Pinatar (Murcia, Spain) by the distinctive yellow coloration of mature fruit [38]. This phenotype was later associated with an elevated proportion of colorless carotenes and ζ-carotene, and reduced downstream β,β-xanthophylls [38, 39]. It is interesting to remark that the concentration of phytoene and phytofluene in ‘Pinalate’ is one of the highest reported among fruits [39, 45]. These carotenes are currently used as nutraceuticals ingredients and in cosmetic products, and their intake has been associated with beneficial effects on human health due to their antioxidant properties and the capacity to reduce the risk of certain cancers (reviewed in [50, 51]). Therefore, understanding the molecular basis of ‘Pinalate’ phenotype may not only provide new clues on critical regulatory steps of carotenoid pathway in *Citrus*, but also valuable information to be used in breeding programs to enhance the content of phytoene and phytofluene in fruits. The carotenoid composition in ‘Pinalate’ fruit suggests that the alteration in the mutant is not associated to the ripening process, as the anomalous carotenoid profile is already manifested from early stages of fruit development (Fig. 2). Moreover, the carotenoid content in leaves also points that ‘Pinalate’ alteration is not restricted to fruit, as was previously proposed [38], since total carotenoids and chlorophylls are substantially reduced in leaves (Fig. 2c). Interestingly, similarly to leaves, green flavedo of ‘Pinalate’ shows reduced carotenoid content (Fig. 2a), which indicates a differential effect of the mutation in chloro- and chromoplasts. This contrasting effect on the carotenoid content in chloroplastic and non-chloroplastic tissues is not unexpected since the regulation and interaction of the carotenoid biosynthetic enzymes depends on plastid type and carotenoids fate in the tissue [7, 52, 53].

Previous works in ‘Pinalate’ proposed a partial blockage in the carotenoid pathway at the level of ζ-carotene desaturation [38, 39, 45]. However, the accumulation of the atypical 9,15,9′-tri-*cis*-ζ-carotene isomer, not previously reported in other citrus fruit, and the absence of neurosporene, that accumulates in ZDS mutants of other plants [33], suggested a defect in Z-ISO, isomerase which catalyzes the *cis-trans* conversion of 15–15’double bond of 9,15,9′-tri-*cis*-ζ-carotene [26, 27, 32], rather than in ZDS. The role of Z-ISO in fruits has been only explored in tomato by VIGS-silencing approach [33] or by characterization of defective tomato mutants (EU FPS DISCO Report https://cordis.europa.eu/project/rcn/110947/reporting/en). In *z-iso* tomato mutants the predominant carotenoid is phytoene (32% of the total content), followed by phytofluene (16%) and ζ-carotene (13%), and the ratio tri-*cis*:di-*cis* is approximately 0.5 [33]. This carotenoid composition resembles very closely that found in ‘Pinalate’ fruit (Figs. 2 and 3).

Light can partially mediate a spontaneous conversion of 9,15,9′-tri-*cis*-ζ-carotene to 9,9′-di-*cis*-ζ-carotene and compensates the alterations in Z-ISO activity [26, 32, 33]. By visual field observations, it was noticed that peel areas of ‘Pinalate’ fruit directly exposed to sunlight developed a light-orange pigmentation in which the proportion of β,β-xanthophylls increased up to 16% (Fig. 3). By contrast, when ‘Pinalate’ fruits were prevented from light the proportion of β,β-xanthophylls was reduced to 1% (Fig. 3). More interestingly, the tri-*cis*:di-*cis* ratio was 2-fold higher in dark- than in light-grown ‘Pinalate’ fruit. All these evidences are in full agreement with those observed in *Z-ISO*-silenced tomato fruit [33]. The phenotype of photosynthetic tissues grown under normal light conditions is less evident and light-exposed leaves of *Z-ISO* mutants only showed a reduced content of carotenoids and chlorophylls associated to pale-green coloration [26, 54]. A similar phenotype was also observed in ‘Pinalate’ young leaves, without other significant effects (Fig. 2c).

The comparative analysis of the transcriptional profiles of the main carotenoid biosynthetic genes in ‘Pinalate’ and ‘Navelate’ also suggest that alterations in Z-ISO may be responsible for the mutant phenotype (Figs. 4 and 8). In ‘Pinalate’, as in the parental fruit, the characteristic up-regulation of *PSY, ZDS1, βLCY2* and *βCHX* genes was associated with the induction of carotenogenesis [11–14], but only in the parental genotype the *PSY* transcript levels correlated well with total carotenoid content [4, 55]. In ‘Pinalate’ flavedo, in contrast with the high content of phytoene at ripe stage, the expression of *PSY* was significantly lower than in the parental (Fig. 4), suggesting a negative feedback regulation of *PSY*. A similar effect was described in knockout *pds* Arabidopsis mutants where the early disruption of the pathway provokes accumulation of phytoene associated with down-regulation of *PSY* and *LCY* [56]. In the pulp, consistent with the lower carotenoid accumulation in this tissue, the relative expression level for all genes was lower than in flavedo [8, 12, 13], and no significant differences in transcript accumulation were detected between both genotypes (Fig. 4). The alterations in gene expression detected in ‘Pinalate’ do not explain per se the abnormal carotenoid profile in the mutant. The biochemical and physiological data strongly suggested a defective Z-ISO in ‘Pinalate’, and analysis of the *Z-ISO* expression resulted in clear differences between mutant and parental genotypes. In ‘Navelate’ fruit *Z-ISO* followed an up-regulated pattern, similar and coordinated with other carotenoid biosynthetic genes, while in ‘Pinalate’ transcript levels of *Z-ISO* were significantly reduced and only a moderate increment was detected in ripe fruit (Figs. 3 and 8). In tomato, *Z-ISO*, together with *PSY1*, is one of the most strongly up-regulated genes during fruit ripening, increasing transcript abundance more than 33- and 20-fold from mature green to breaker and ripe stage, respectively [33, Sol Genomics Network expression database http://tea.solgenomics.net/expression_viewer/output gene Solyc12g098710].

It is remarkable that in ‘Navelate’ leaves the *Z-ISO* expression was as high as in ripe flavedo, suggesting an important role of this enzyme in the biosynthesis of carotenoids in chloroplasts, likely involved in carotenoid biosynthesis modulation under stress conditions [26].

As in other plants, *Z-ISO* is found in the sweet orange genome as single-copy gene showing a well conserved structure (Fig. 5). Modelling the sweet orange Z-ISO predicted an integral membrane protein with seven transmembrane domains and the three residues, H158, C271 and H274, were located in the 3D model in positions compatible with the proposed mechanism of ligand heme rearrangement [27]. The functionality of the sweet orange Z-ISO was confirmed by heterologous expression in *E.coli* (Fig. 7).

The genomic sequence of ‘Pinalate’ *Z-ISO* revealed a single T insertion at exon 2, resulting in the generation of three different *Z-ISO* transcripts (Fig. 6). Since the sequencing showed that the mutation is in heterozygosity (Additional file 3: Figure S3), it was expected the *WT* transcript in ‘Pinalate’, but two more additional sequences, so-called *P6* and *P5*, encoding truncated Z-ISO proteins with no activity in in vitro assays, were identified (Figs. 6 and 7). In line with previous studies, shorter *Z-ISO* transcripts with one less transmembrane domain encoded a non-functional protein, suggesting that this part of the protein is essential for the isomerase activity [27]. The 1:1 proportion of *WT* and altered (*P5* and *P6*) sequences in ‘Pinalate’ also agrees with the heterozygous nature of the mutation (Additional file 3: Figure S3). In view of this situation, explaining of the phenotype of ‘Pinalate’ is not straightforward because null mutations in *Z-ISO* in other species are recessive [26, 32]. The cDNA data indicated that the *WT* allele is transcribed in ‘Pinalate’ and comprises about half of *Z-ISO* transcripts. Therefore, ‘Pinalate’ displays a definite phenotype expected from a lesion in Z-ISO with some leakiness that is manifested by synthesis of xanthophylls. A possible explanation could be dominant-negative effects by truncated Z-ISO polypeptides that interfere with the functioning of the *WT* protein. Moreover, the reduced transcript level of *Z-ISO* in ‘Pinalate’ may be explained by a nonsense-mediated mRNA decay mechanism, which detects and rapidly degrades transcripts containing premature termination codons as reported for *CRTISO* gene in tomato *tangerine*^*3002*^ mutant [57].

One interesting feature is that the predominant carotenoid in ‘Pinalate’ mutant is phytoene, product of PSY activity, instead of the tri-*cis*-ζ-carotene isomer, the substrate of Z-ISO. This proportion of carotenes fits with the model of three metabolic units proposed by Fantini et al. (2013) [33] in tomato. In this model, PDS and Z-ISO are part of a functional unit and a defect in one of the components, as in this case Z-ISO, would result in the preferential accumulation of phytoene, the substrate of the unit. Therefore, although carotenoid composition in ripe sweet orange fruit is very different to that found in tomato, the biosynthesis of carotenoids in chromoplasts of both species seems to share common regulatory mechanisms and similar structural organization of the early steps in multi-enzymatic complexes.

## Conclusions

The yellow pigmentation of the sweet orange mutant ‘Pinalate’ is due to a reduction in β,β-xanthophylls and derived apocarotenoids. Analysis of carotenoid composition in fruit tissues identified the presence of six different ζ-carotene isomers in ‘Pinalate’, including the 9,15,9′-tri- *cis*-ζ-carotene, which was not previously reported in other citrus fruits. The dynamic changes in carotenoid profile in ‘Pinalate’ fruit tissues during ripening strongly suggests an impairment in Z-ISO activity, a recently discovered 15-*cis*-isomerase involved in the transformation of 9,15,9′-tri- *cis*-ζ-carotene to 9,9′-di-*cis*-ζ-carotene. The transcriptional analysis of main carotenoid biosynthetic showed no significant differences between ‘Pinalate’ and ‘Navelate’ genotypes that explain the mutant phenotype. Functional assays indicate that *Z-ISO* gene from sweet orange encodes a bona fide 15-*cis*-isomerase. The isolation of ‘Pinalate’ *Z-ISO* genomic sequence revealed a single nucleotide insertion in *Z-ISO* exon 2 in heterozygosis. This point mutation results in two additional alternative transcripts with a premature stop codons and non-functional proteins. The wild-type transcript version was also identified in ‘Pinalate’ fruit but a nonsense-mediated mRNA decay mechanism seems to operate since the expression of *Z-ISO* in ‘Pinalate’ is highly down-regulated compared to the parental. Remarkably, the effect of the mutation is less evident in chloroplastic tissues, suggesting that the role of this activity can be partially dispensable in photosynthetic tissues. In summary, the molecular basis of the altered carotenoid biosynthesis in the yellow ‘Pinalate’ sweet orange mutant has been identified as a single nucleotide insertion in *Z-ISO* gene, producing a massive accumulation of early upstream carotenes and revealing the crucial role of this activity in citrus carotenogenesis and fruit pigmentation.

## Methods

### Plant material and treatments

Plant material used for the experiments was collected from adult trees of sweet orange (*Citrus sinensis* L. Osbeck) cv. ‘Navelate’ and ‘Pinalate’ from the Citrus Germplasm Bank grown at Instituto Valenciano de Investigaciones Agrarias (Moncada, Valencia, Spain) (http://www.ivia.gva.es/es/banco-de-germoplasma-de-citricos) [38]. Twenty Peel and pulp tissue from at least 15 fruits was collected at three developmental stages: immature green, harvested in July between 90 and 100 DAA (days after anthesis); breaker, harvested in October between 170 and 180 DAA; ripe, harvested in January between 280 and 300 DAA (Fig. 2). Fruits were selected for color and size uniformity, and absence of any lesion or injury. Immediately after harvest, flavedo tissue (outer and colored peel layer of the fruit) was collected with a scalpel carefully separating it from the albedo (white spongy mesocarp), and pulp juice vesicles were excised from central segments. Fully expanded young leaves (less than four months old) from the same orange trees were also collected. All the plant material was immediately frozen in liquid nitrogen, ground to a fine powder and stored at − 80 °C until analysis.

To explore the effect of light on carotenoid composition in ‘Pinalate’ fruit peel, immature green fruits (July) were covered (dark grown) with black plastic bags with end-bottom open to allow gas exchange [46] and harvested in December. For comparison, non-covered ‘Pinalate’ fruits (light grown) were harvested in the same date. To maximize the effect of light exposure light grown fruits were chosen from the external canopy of the tree that was oriented towards the South which is the sunniest side. Two replicates consisting of 15 fruits were harvested from covered (dark growing conditions) and (light growing conditions) oranges and flavedo tissue was collected and stored as described above.

### Carotenoid extraction and quantification from fruit tissues and leaf

Carotenoids from flavedo, pulp and leaf tissues were extracted as previously described [38, 39] with slight modifications. Briefly, freeze ground material of flavedo (0.5 g), pulp (2 g) or leaf (0.25 g) was extracted essentially as previously described [14]. The carotenoid extracts were analyzed by HPLC in a Waters 600E pump and 2998 photodiode array detector (PAD) as described [18]. Carotenoid pigments were separated by HPLC using a C_30_ carotenoid column (250 × 4.6 mm, 5 μm) coupled to a C_30_ guard column (20 × 4.0 mm, 5 μm; YMC, Teknochroma, Spain) with ternary gradient elution of MeOH, water and methyl *tert*-butyl ether [58]. Carotenoids were identified by their retention time, absorption and fine spectra [26, 33, 59, 60]. For each elution a Maxplot chromatogram was obtained and carotenoid contents were calculated using calibration curves of zeaxanthin (Extrasynthese), β-carotene (Sigma) for α- and β-carotene, β-cryptoxanthin (Extrasynthese), lutein (Sigma), antheraxanthin (CaroteNature), violaxanthin (CaroteNature) for violaxanthin and neoxanthin isomers, and β-apo-8′-carotenal (Extrasynthese) for β-citraurin. Phytoene, phytofluene and ζ-carotene were previously purified as described [61] by thin layer chromatography from carotenoid extracts of ‘Pinalate’ orange fruit and the corresponding calibration curves performed [39]. The concentration of individual carotenoids are expressed as micrograms per g of sample on fresh weight basis (μg g^− 1^ FW).

Samples were extracted at least twice and each analytical determination was replicated. All operations were carried out on ice under dim light to prevent photodegradation, isomerisation and structural changes of carotenoids.

### Isolation of genomic and cDNA sequences of the *Z-ISO* gene in ‘Navelate’ wild-type and ‘Pinalate’ mutant

Genomic DNA from immature fruits and leaves of *C. sinensis* ‘Navelate’ (wild-type cultivar) and ‘Pinalate’ (mutant) was isolated using DNeasy Plant Mini Kit (Qiagen) and was used to amplify the *Z-ISO* genomic sequences from both genotypes by PCR. The amplification was carried out using the primers MJ302F (5′- ATGAGCAGCAGTAGTTGTCTTCTTCTC-3′) and MJ415R (5′- GCTTCTTTAATTCAAAGTTTGGGTTGCTTC-3′) designed based on the orange1.1g017272m.g gene sequence (www.phytozome.com), and AccuPrime™Taq DNA Polymerase High Fidelity (Invitrogen). The cycling program consisted of 2 min at 94 °C, then 35 cycles of 15 s at 94 °C for denaturation, 30 s at 57 °C for annealing and 3 min at 68 °C for extension. The RNA isolation and cDNA synthesis from flavedo, pulp and leaf tissues were performed essentially as described in [62]. Briefly, total RNA was isolated by using RNeasy Plant Mini Kit (Qiagen) and subsequently treated with DNase I (DNA free, DNase treatment and removal, Ambion). The transcripts present in 5 μg of total RNA were reverse-transcribed using the Superscript III reverse transcriptase (Invitrogen) in a total volume of 20 μL. One μL of a tenfold diluted first-strand cDNA was used for the amplification of *Z-ISO* cDNA by PCR. The cycling program consisted of 2 min at 94 °C, then 35 cycles of 15 s at 94 °C for denaturation, 30 s at 57 °C for annealing and 1 min at 68 °C for extension. Two independent cloning experiments were carried out using PCR products amplified from the cDNA of the peel and pulp of ‘Navelate’ and ‘Pinalate’ at breaker stage, and the genomic DNA from immature green fruits and leaves. The fragments were cloned into pGEM®-T (Promega Corporation, Madison, WI, USA) and at least 10 or 71 independent clones were sequenced per genotype for the genomic or cDNA amplification, respectively.

### Functional analysis of *Citrus Z-ISO* in *Escherichia coli*

Full length cDNAs of *Z-ISO* isolated from ‘Navelate’ and ‘Pinalate’ were cloned in the plasmid vector pGEM®-T (Promega Corporation, Madison, WI, USA). These plasmids were transfected into *E. coli* strain XL1-Blue carrying the ζ-carotene-producing plasmid pZETA, which carries the genes *crtB* and *crtE* from *Pantoea agglomerans* and *crtP* from the cyanobacterium *Synechococcus elongatus* PCC7942 in the plasmid vector pACYC184, or the prolycopene-producing pPROLYCOPENE [63]. Cells of *E. coli* carrying pZETA or pPROLYCOPENE vectors alone served as a control.

Two millilitres of overnight suspension culture of *E. coli* cells were inoculated into 100 mL of Luria-Bertani medium in a 500 mL flask with antibiotics chloramphenicol (34 mg L^− 1^) and ampicillin (50 mg L^− 1^). Cells were cultured for 8 h at 37 °C in the dark and then induced with 10 mM isopropylthio-*β*-galactoside, followed by 40 h of slow shaking (100 rpm) at room temperature and an additional 2 day without shaking prior to carotenoid extraction. Bacterial cells were collected by centrifugation and the pellet was extracted in acetone until loss of color. The acetone extracts were dried under a stream of N_2_ and re-dissolved in 500 μl acetone for HPLC analysis.

For testing the effect of light, flasks were covered with aluminum foil; otherwise the light intensity was ca. 50 μE m^− 2^ s^− 1^. Carotenoids were analyzed by HPLC using a Waters system consisting of Waters 600 pump, Waters 996 PAD and Waters 717 plus Autosampler as previously describe [64].

### Gene expression analysis

Gene expression analyses were performed following the Minimum Information for publication of Quantitative real-time PCR Experiments (MIQE) guidelines [65]. Quantitative real-time PCR was carried out on a LightCycler 480 instrument (Roche) following conditions described in [66]. The RNA isolation and cDNA synthesis from flavedo, pulp and leaf tissues were performed essentially as described in [62] and mentioned above in the section entitled isolation of genomic and cDNA. The absence of DNA contamination was checked in all samples by performing a no-reverse transcription assay which consisted of a PCR with each RNA sample using the citrus *ACTIN* primers The primers employed for the amplification of the *PSY*, *PDS*, *βLCY1*, *βLCY2*, *βCHX, ZEP* were described in [58], for *ZDS1*, *ZDS2*, *ZDS3* in [46] and for Z*-ISO* were *MJ283F* (sense, 5′-GCAGCGTCACTGGGTTTAAT-3′) and *MJ284R* (antisense, 5′- GTTCGCCTCTTCACAGCTTC-3′) which amplify a region in the exon 4. The cycling protocol conditions used for all genes are essentially as described previously in [67]. The calculated expression levels were relative to values of an external reference sample (flavedo from ‘Navelate’ fruit at mature green stage harvested in September) using the Relative Expression Software Tool [68]. Normalization was performed using the expression levels of the *ACTIN* gene [62]. Results were the average of 4 independent replicates.

To test for significant differences on transcript levels between samples, a pair-wise fixed reallocation randomization test was applied (*P* ≤ 0.01) [68].

## Supplementary information

**Additional file 1: Figure S1.** Spectra of ζ-carotene isomers identified in chromatograms of ‘Pinalate’ carotenoid extracts. Z1, 9,15,9′-tri-*cis*-ζ-carotene; Z2, ζ-carotene isomer; Z3, ζ-carotene isomer; Z4, 9,9′-di-*cis*-ζ-carotene; Z5, ζ-carotene isomer; Z6, ζ-carotene isomer.

**Additional file 2: Figure S2.** Representative MaxPlots chromatograms obtained from pulp carotenoids extracts of ‘Navelate’ (parental) and ‘Pinalate’ (mutant).; U1, unknown (wavelength absorbance spectrum: 401,416,446); U2, unknown (426,450); U3, unkown (320,356,377); N, Neochrome; Nx, neoxanthin; V1, all-*trans*-violaxanthin; V2, 9-*cis*-violaxanthin; L, lutein; Zx, zeaxanthin; A, antheraxanthin;P1, 15-*cis*-Phytoene; Pf1, Phytofluene isomer; Pf2, Phytofluene isomer2; Cx, β-Cryptoxanthin; Z1, 9,15,9′-tri-*cis*-ζ-carotene; Z2, ζ-carotene isomer; Z3, ζ-carotene isomer; Z4, 9,9′-di-*cis*-ζ-carotene; Z5, ζ-carotene isomer; Z6,ζ-carotene isomer; BC, β-carotene.

**Additional file 3: Figure S3.** Direct sequencing of *Z-ISO* genomic DNA from ‘Pinalate’ amplified by PCR. The nucleotide sequencing with a reverse primer in *Z-ISO* trace up to the T insertion site (indicated with an *) showing identical sequence between WT and ‘Pinalate’ alleles (bold letters). From the T insertion and on, the sequence trace became blurred in ‘Pinalate’ genomic due to a frameshift in one allele.

**Additional file 4: Figure S4.** Functional analysis of citrus Z-ISO in *E.coli* pPROLYCOPENE strain. Carotenes composition in extracts from *E. coli* with pPROLYCOPENE plasmid and C Z-ISO from ‘Navelate or ‘Pinalate’ (mutant) P5 and P6 cDNAs. *E. coli* with pPROLYCOPENE alone was used as control. Carotenoid analysis in *E. coli* cells was determined by HPLC-PAD as described in Material and Methods section.

## Data Availability

All data supporting the findings is contained in the manuscript and its supplementary files.

## Abbreviations

ABA: Abscisic acid
DAA: Days after anthesis
FW: Fresh weight
PDS: Phytoene desaturase
PSY: Phytoene synthase
ZDS: ζ-carotene desaturase
Z-ISO: 15-*cis*-ζ-carotene isomerase
βCHX: Non-heme β-carotene hydroxylase
βLCY1: Lycopene β-cyclase 1
βLCY2: Chromoplast-specific lycopene β-cyclase2
εLCY: Lycopene ε-cyclase
