# Supplementary material for: A mutant allele of ζ-carotene isomerase (Z-ISO) is associated with the yellow pigmentation of the “Pinalate” sweet orange mutant and reveals new insights into its role in fruit carotenogenesis
Source: BMC Plant Biol. 2019 Nov 4;19:465. doi: 10.1186/s12870-019-2078-2 (PMC6829850; doi:10.1186/s12870-019-2078-2)
Supplement: Supplementary file 1 — Additional file 1: Figure S1. Spectra of ζ-carotene isomers identified in chromatograms of ‘Pinalate’ carotenoid extracts. Z1, 9,15,9′-tri-cis-ζ-carotene; Z2, ζ-carotene isomer; Z3, ζ-carotene isomer; Z4, 9,9′-di-cis-ζ-carotene; Z5, ζ-carotene isomer; Z6, ζ-carotene isomer. [file 12870_2019_2078_MOESM1_ESM.pdf]

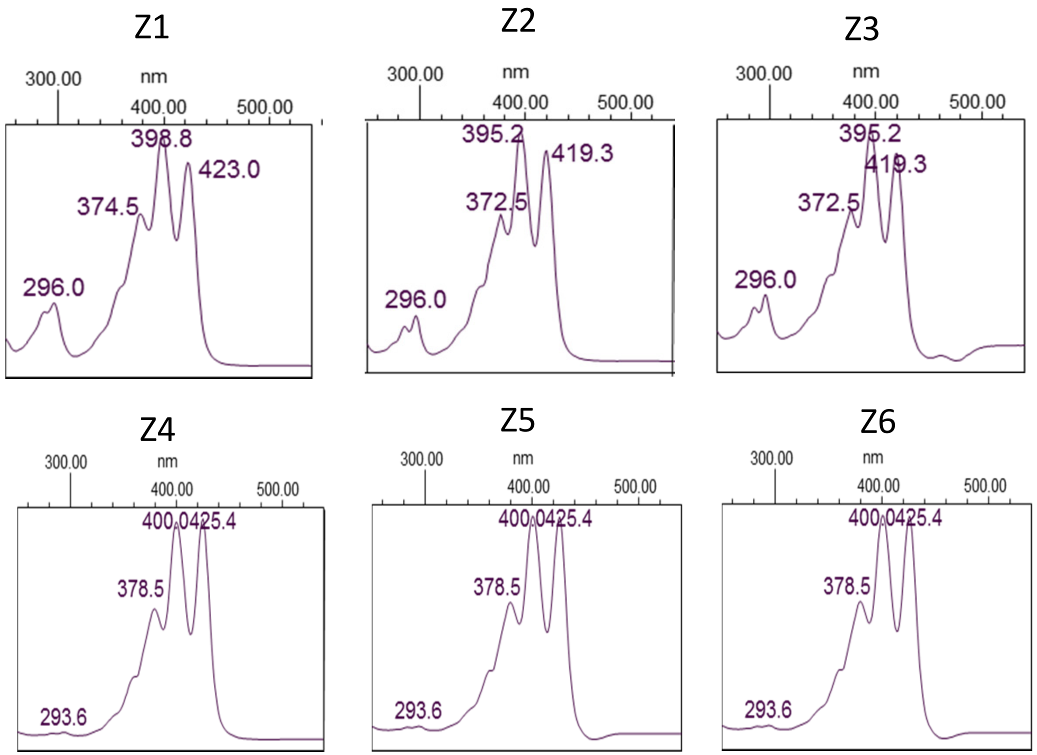

Figure S1. Spectra of ζ-carotene isomers identified in chromatograms of Pinalate carotenoid extracts. Z1, 9,15,9´-tri-*cis*–ζ-carotene; Z2, ζ-carotene isomer; Z3, ζ-carotene isomer; Z4, 9,9´-di-*cis*–ζ-carotene; Z5, ζ-carotene isomer; Z6,ζ-carotene isomer.
