# Supplementary material for: A mutant allele of ζ-carotene isomerase (Z-ISO) is associated with the yellow pigmentation of the “Pinalate” sweet orange mutant and reveals new insights into its role in fruit carotenogenesis
Source: BMC Plant Biol. 2019 Nov 4;19:465. doi: 10.1186/s12870-019-2078-2 (PMC6829850; doi:10.1186/s12870-019-2078-2)
Supplement: Supplementary file 2 — Additional file 2: Figure S2. Representative MaxPlots chromatograms obtained from pulp carotenoids extracts of ‘Navelate’ (parental) and ‘Pinalate’ (mutant).; U1, unknown (wavelength absorbance spectrum: 401,416,446); U2, unknown (426,450); U3, unkown (320,356,377); N, Neochrome; Nx, neoxanthin; V1, all-trans-violaxanthin; V2, 9-cis-violaxanthin; L, lutein; Zx, zeaxanthin; A, antheraxanthin;P1, 15-cis-Phytoene; Pf1, Phytofluene isomer; Pf2, Phytofluene isomer2; Cx, β-Cryptoxanthin; Z1, 9,15,9′-tri-cis-ζ-carotene; Z2, ζ-carotene isomer; Z3, ζ-carotene isomer; Z4, 9,9′-di-cis-ζ-carotene; Z5, ζ-carotene isomer; Z6,ζ-carotene isomer; BC, β-carotene. [file 12870_2019_2078_MOESM2_ESM.pdf]

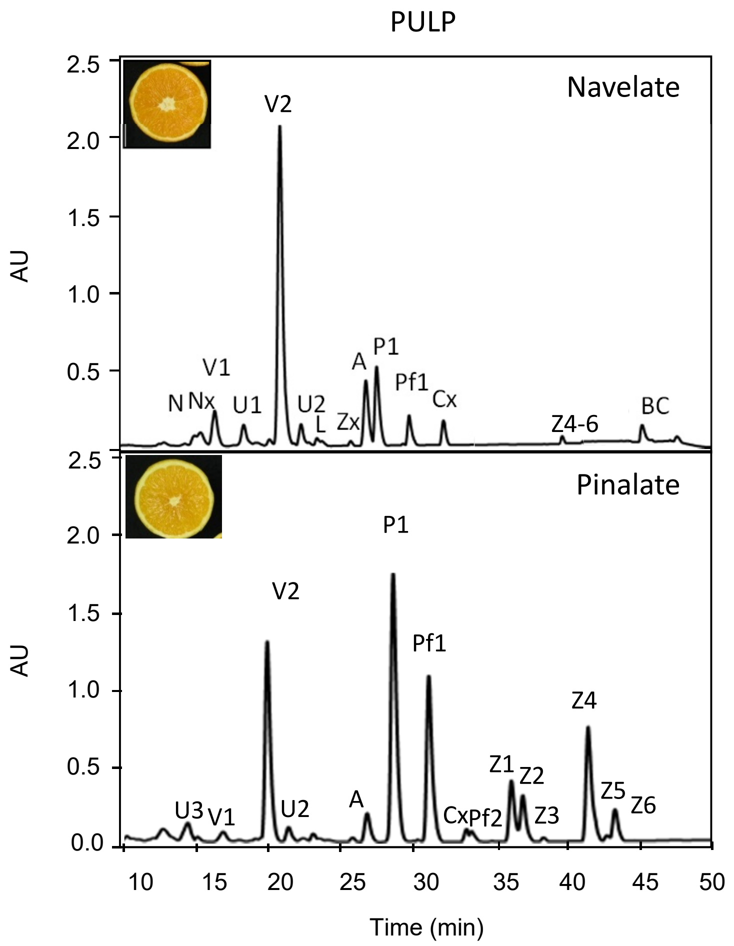

Figure S2. Representative MaxPlots chromatograms obtained from pulp carotenoids extracts of Navelate (parental) and Pinalate (mutant).; U1, unknown 401, 416, 446; U2, unknown 426,450; U3, 320,356,377; N, Neochrome s,432,458; Nx, neoxanthin; V1, all-*trans*-violaxanthin; V2, 9-*cis*-violaxanthin; L, lutein; Zx, zeaxanthin; A, antheraxanthin;P1, 15-*cis*-Phytoene; Pf1, Phytofluene isomer; Pf2, Phytofluene isomer2; Cx, β-Cryptoxanthin; Z1, 9,15,9´-tri-*cis*–ζ-carotene; Z2, ζ-carotene isomer; Z3, ζ-carotene isomer; Z4, 9,9´-di-*cis*–ζ-carotene; Z5, ζ-carotene isomer; Z6,ζ-carotene isomer; BC, β-carotene.
