# Supplementary material for: A mutant allele of ζ-carotene isomerase (Z-ISO) is associated with the yellow pigmentation of the “Pinalate” sweet orange mutant and reveals new insights into its role in fruit carotenogenesis
Source: BMC Plant Biol. 2019 Nov 4;19:465. doi: 10.1186/s12870-019-2078-2 (PMC6829850; doi:10.1186/s12870-019-2078-2)
Supplement: Supplementary file 3 — Additional file 3: Figure S3. Direct sequencing of Z-ISO genomic DNA from ‘Pinalate’ amplified by PCR. The nucleotide sequencing with a reverse primer in Z-ISO trace up to the T insertion site (indicated with an *) showing identical sequence between WT and ‘Pinalate’ alleles (bold letters). From the T insertion and on, the sequence trace became blurred in ‘Pinalate’ genomic due to a frameshift in one allele. [file 12870_2019_2078_MOESM3_ESM.pdf]

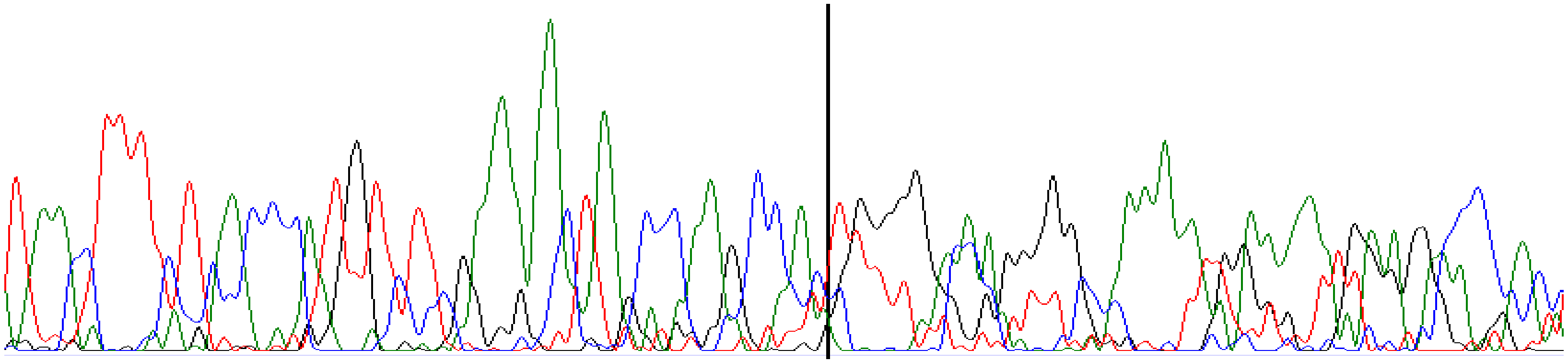

TAACTTTCTCACCCATGTCTCGAAGACTAGCCAAGCCANNNNgNNNNCNGNNNNNTNNNNNT

*

NNNNNANNNNNCNGNNNNcNNNN**TGGCTTGGCTAGTCTTCGAGACATGGGTGAGAAAGTTA**TTGGTGCACGTGCT

TATCGTGTTCTGTTCGCAGGAGTATCTCTTCCATTAGCTGTTAGTACTATTG ← rev primer

Figure S3. Direct sequencing of genomic DNA of Z-ISO from Pinalate amplified by PCR. The nucleotide sequencing with a reverse primer in Z-ISO trace up to the T insertion site (indicated with an *) showing identical sequence between both alleles (bold letters). From the T insertion and on, the sequence trace became blurred in Pinalate genomic due to a frameshift in one allele.
