# Supplementary material for: A mutant allele of ζ-carotene isomerase (Z-ISO) is associated with the yellow pigmentation of the “Pinalate” sweet orange mutant and reveals new insights into its role in fruit carotenogenesis
Source: BMC Plant Biol. 2019 Nov 4;19:465. doi: 10.1186/s12870-019-2078-2 (PMC6829850; doi:10.1186/s12870-019-2078-2)
Supplement: Supplementary file 4 — Additional file 4: Figure S4. Functional analysis of citrus Z-ISO in E.coli pPROLYCOPENE strain. Carotenes composition in extracts from E. coli with pPROLYCOPENE plasmid and C Z-ISO from ‘Navelate or ‘Pinalate’ (mutant) P5 and P6 cDNAs. E. coli with pPROLYCOPENE alone was used as control. Carotenoid analysis in E. coli cells was determined by HPLC-PAD as described in Material and Methods section. [file 12870_2019_2078_MOESM4_ESM.pdf]

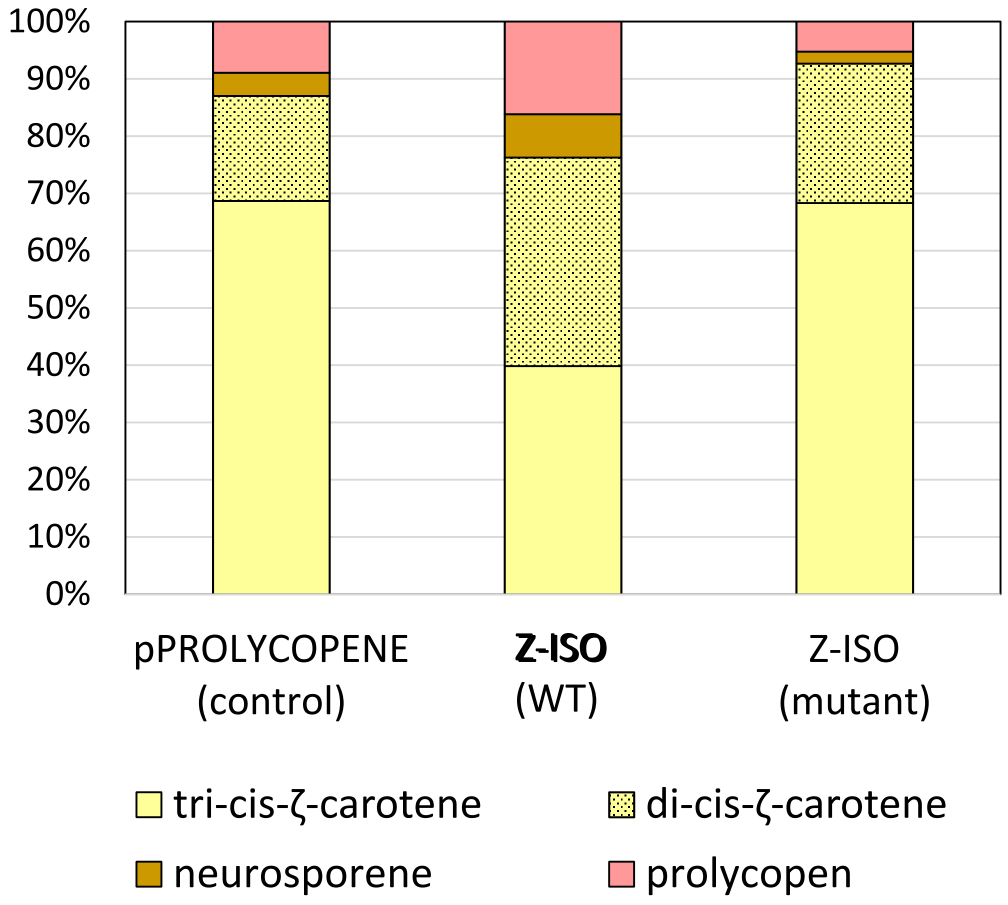

Figure S4. Functional analysis of Citrus Z-ISO in *E.coli* pPROLYCOPENE strain. Carotenes composition in extracts from *E. coli* with pPROLYCOPENE plasmid and Citrus Z-ISO from WT or Pinalate (mutant) P5 and P6 cDNAs. *E. coli* with pPROLYCOPENE alone was used as control. Carotenoid analysis in *E. coli* cells was determined by HPLC-PAD as described in Material and Methods section.
